# Knowledge and practices towards malaria amongst residents of Bushbuckridge, Mpumalanga, South Africa

**DOI:** 10.4102/phcfm.v3i1.257

**Published:** 2011-07-12

**Authors:** Khumbulani W. Hlongwana, Alpheus Zitha, Aaron M. Mabuza, Rajendra Maharaj

**Affiliations:** 1 Malaria Research Programme, South African Medical Research Council, Durban, South Africa; 2 Malaria Control Programme, Department of Health and Social Services, South Africa

## Abstract

**Background:**

Malaria remains one of the greatest public health challenges worldwide and it is amongst the top killers in sub-Saharan Africa. There is however, a general scepticism about the accuracy of Health Management Information Systems (HMIS) in recording all the episodes of malaria in Africa. Given the importance of community knowledge of malaria, its signs and symptoms, as well as prompt treatment-seeking behaviour, the study assessing adult residents’ knowledge and practices in Bushbuckridge provided much needed insights into the Malaria Control Programme (MCP).

**Objectives:**

The objectives of this study were to determine the adult residents’ knowledge and practices towards malaria in Bushbuckridge, Mpumalanga Province, South Africa.

**Method:**

The study was undertaken as a descriptive cross-sectional survey in Bushbuckridge in August 2008. Six hundred and two (602) household heads or their proxies from the randomly selected households in 20 localities were interviewed (one household member per household), using a structured field-piloted questionnaire.

**Results:**

Approximately 93% of the respondents had heard about malaria, 84.6% of whom correctly associated it with mosquito bites. The health facility (29.1%) and radio (19.8%) were the main sources of malaria information. Knowledge of signs and symptoms was low, whilst treatment-seeking intention at the health facility was high (99%) with 82% of which would be carried out promptly. Survey data showed an indoor residual spraying (IRS) coverage of approximately 70% and a good understanding of the reasons for spraying. Walls were re-plastered infrequently and no evidence was established linking it to the removal of insecticide marks on the wall.

**Conclusion:**

The study revealed not only that householders possessed an adequate knowledge of malaria, but also that they had positive malaria treatment-seeking intentions. Their knowledge of malaria signs and symptoms was inadequate and required attention. Whilst IRS coverage needed some improvements, the reasons for IRS were well known.

## Introduction

### Setting

Malaria remains one of the world's greatest public health challenges,^1^ killing over one million people per year,^2^ and up to 500 million clinical malaria cases occur globally.^3, 4^ Whilst Africa accounts for over 90% of the disease burden worldwide,^5, 6, 7, 8, 9^ sub-Saharan Africa is the worst afflicted malaria region,^10, 11, 12, 13^ and malaria is one of the top killers.^14^ Other authors^8^ insist that malaria episodes in Africa are underestimated and many cases never reach health facilities, and are therefore not captured by the health management information systems (HMIS). Notably, the flow of malaria cases to health facilities is likely to be affected by the treatment-seeking behaviour as well as the amount and quality of malaria health education provided to the community. Human migration across national borders is another critical factor which is likely to compromise the effectiveness of malaria-control interventions,^15, 16^ especially malaria information, education and communication (IEC) and promptness in treatment-seeking practices. Cross-border movement does not only pose a risk of malaria parasites being imported into the country, but some people may arrive *after* IEC activities have been conducted, so the monitoring of IEC impact can become very difficult.

Despite the documentation of numerous health compromising factors, some studies have emphasised the value of adequate knowledge of malaria in order to ensure that people apply preventive measures, and seek prompt and appropriate treatment for themselves and their dependants.^9, 17^ It is necessary, therefore, that people's knowledge and practices with regard to malaria is regularly assessed and promoted. Nevertheless, no malaria knowledge and practices studies were previously conducted in Bushbuckridge. The main rationale for this study was to investigate and describe the local adult residents’ understanding of malaria transmission, their recognition of signs and symptoms, their perceptions of cause, treatment and preventive practices, and indoor residual spraying (IRS), in view of the migratory trend between Mozambique and Bushbuckridge. The ultimate goal was to produce informative results which could be used to improve the educational materials designed for use in IEC intervention strategy.

In South Africa, malaria transmission is seasonal and restricted to the north-eastern border areas with Mozambique, Swaziland and Zimbabwe.^18, 19^ Approximately 4.9 million (10%) of South Africa's total population are at risk of contracting malaria^20^ and annual national malaria cases were estimated at 32 530 in the year 2006.^21^ *Anopheles arabiensis* remains the only confirmed vector plaguing the country; following the elimination of *Anopheles funestus* through years of IRS with dichlorodiphenyltrichloroethane (DDT).^19^ *Plasmodium falciparum* is the most prevalent parasite, accounting for approximately 95% of all malaria cases in South Africa.^22^ IRS with DDT and K-Othrine (a pyrethroid insecticide), complemented with effective case management (definitive diagnosis and effective treatment), disease surveillance and malaria health promotion are the major control strategies employed in Bushbuckridge. IRS is implemented once a year, whereby K-Othrine is applied on smooth-surface painted inner house walls (usually referred to as western structures) and DDT is applied on rough-surface unpainted inner house walls (usually referred to as traditional structures). Artemisinin combination therapies (ACT) and quinine remain the first and second-line treatment drugs, respectively.

### Significance of the study

Malaria in South Africa remains a public health challenge as in various other countries of the world. There are two striking factors about this study, that is, the timing of the study and the geographical location of the study site. The timing of the study is significant, because it was carried out after South Africa was declared ready to eliminate malaria. Geographically, the study area is in close proximity to Mozambique, which is still in the control stage of malaria, whilst there is documented evidence of high cross-border movements between Mozambique and Bushbuckridge. Most importantly, results of the study were to be used directly by the Malaria Control Programme to improve its intervention strategies, especially with regard to malaria information, education and communication (IEC).

## Methods

### Setting

**FIGURE 1:**
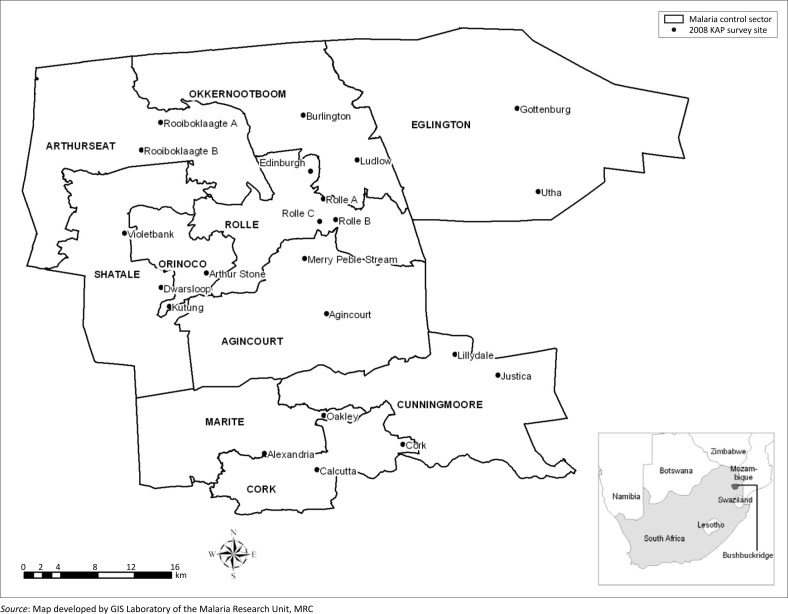
The location of study sites in Bushbuckridge and the position of the subdistrict within the country.

Mpumalanga Province, the third smallest populated province in South Africa, occupies 6% of the country's surface area and lies between Mozambique in the east, Swaziland and KwaZulu-Natal Province in the south, Limpopo Province in the north and Gauteng Province in the west.^23^ Bushbuckridge subdistrict comprises 67 localities (villages) that are clustered into 10 Sectors as demarcated by the Malaria Control Programme for operational purposes (Figure 1).^25^ It extends over an area of 2 400 km^2^,^25^ is populated by approximately 700 000 people and has more than 110 000 households.^21, 25, 26^ Approximately 30% of the adult population originate from Mozambique,^27^ the majority of whom have settled in Bushbuckridge during the Mozambican civil wars of the 1980s.^28, 29^ The Shangaan (vernacular) language and culture shared by the migrants and the local inhabitants, played a paramount role in Mozambican migrants’ choice of Bushbuckridge.^29^

**FIGURE 2:**
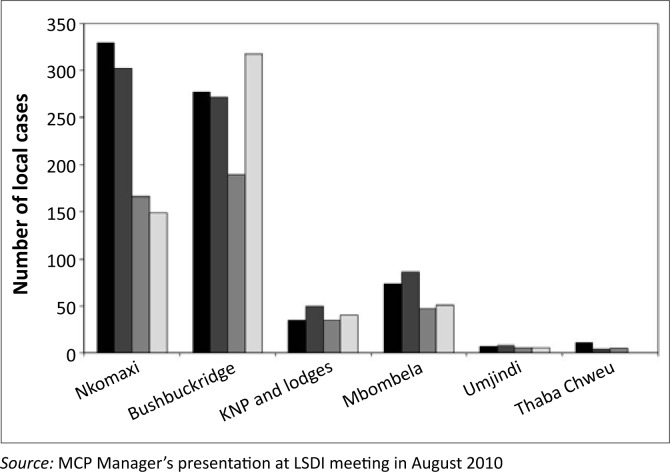
Local malaria cases by season and by Municipal Areas in Mpumalanga Province (black bar denotes 2006–2007, dark grey bar for 2007–2008, light grey bar for 2008–2009 and white bar for 2009–2010 malaria seasons).

Polzer^28^ has documented the systematic integration of Mozambican refugees in Bushbuckridge extensively, as well as regular cross-border movements, from a social-connectivity point of view. From a malaria-epidemiological point of view, however, cross-border human movement provides a critical dimension, given the geographical proximity of Mozambique to South Africa and the frequency of cross-border movements. The upsurge of malaria cases in Bushbuckridge during the 2009–2010 malaria season is a cause of major concern, given the maturing efforts to eliminate malaria in South Africa (Figure 2).^30^ Another important dimension is the two countries’ positions in the malaria-control continuum. South Africa, for example, is already preparing for malaria elimination after being earmarked and declared by the African Union (AU) and the Southern Africa Development Community (SADC) as one of the countries ready for malaria elimination, alongside Swaziland, Botswana and Namibia.^31, 32^ Mozambique, on the other hand, is still at the control stage of malaria.

**FIGURE 3:**
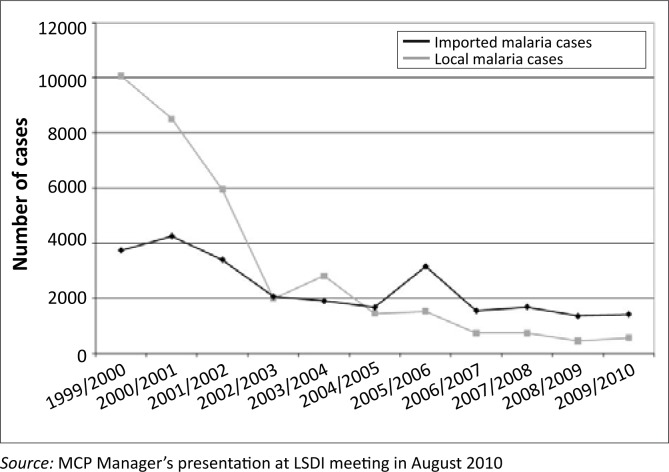
Trends in local and imported malaria cases over a period of 10 years in Mpumalanga Province (the black line denotes imported cases and the grey line denotes local cases).

It appears that imported cases will continue to pose a serious challenge to malaria- control efforts in South Africa and Bushbuckridge in particular. This assertion is confirmed by the recorded trend of malaria cases in Mpumalanga Province (Figure 3),^32^ whereby, over time, the number of imported cases has exceeded that of local cases. There is already documented evidence elsewhere that confirms that the stream of incoming malaria cases from the neighbouring countries compromise the effectiveness of malaria-control interventions.^15^ The complexity of the cross-border movements in Bushbuckridge is further entrenched through intermarriages, a phenomenon that has implications on malaria-control interventions, especially on the eve of a malaria-elimination programme in South Africa.

### Design and data collection

The study was conducted as a descriptive cross-sectional survey. Six locally recruited and competently trained fieldworkers administered a standardised pretested structured questionnaire to 602 adult respondents over a period of 2 weeks (August 2008). The questionnaire (translated from English to XiTsonga and SePedi) included closed-ended, partially closed and open-ended questions (see Appendix B: Questionnaire in English). Study participants were household heads and/or their proxies older than 18 years.

If it is assumed that the number of households has increased from 110 000 to 130 000, a sample size can be calculated based on the systematic conformal sampling technique (refer to the following equations), using the error margin of 4% and the confidence level of 95%, to result in a minimum sample size of 598 households.

x=Z(c/100)2r(100-r)

$$n=Nx/\left(\left[N-1\right]E^{2}+x\right)$$

$$E=\sqrt{}\left(\left[N-n\right]x/n\left[N-1\right]\right)$$

The variables are: *n*, the sample size; *N*, the population size; *E*, the margin of error; *r*, the fraction of the response that we are interested in (with a normal distribution of 50%), and *Z*(*c*/100), the critical value for the confidence level.

The overall sampling strategy was a multistage process, whereby two localities were randomly selected in each sector before disproportionately identifying approximately 30 households (one participant per household) in each locality. Households were identified through systematic sampling with a random start, whereby every tenth household was selected until the required sample size was acquired. Ethics approval was granted by the Mpumalanga Provincial Research and Ethics Committee.

### Analysing

Survey data were entered directly into an electronic data collection system using Microsoft Access, and checked for accuracy prior to analysis. Data were imported from Access, and analysis was performed using the Epi-info (version 3.3.2) statistical software programme. Firstly, a descriptive statistical analysis was carried out. A bivariate analysis (chi-squared test) was applied to compare proportions and to generate Odds Ratios. The statistical significance of the association between variables was assessed using 95% Confidence Intervals (CI). In addition to textual descriptions, results were summarised in the form of Tables and Figures.

## Results

### Characteristics of study population

Three-quarters of the heads of households or their proxies who participated in the study were female (*n* = 451, 75.0%). Approximately one-third of the respondents were under 30 years old, with the average median ages of 35 (s.d. = 14) and 38 years (s.d. = 17) for female and male participants respectively. A number of households (*n* = 262, 43.5%) had family sizes of 4–6 members. Fewer households (*n* = 32, 5.3%) had family members exceeding nine, whilst households with 1–3 and 7–9 family members were 27.1% (*n* = 163) and 24.1% (*n* = 145), respectively. More than half of the respondents (58.8%) had completed secondary education, whilst 20.6% had no formal education. About two-thirds of the respondents were either unemployed or housewives. Self-reported data showed that 11.5% of the respondents had been tested for malaria within the 12 months prior to the survey. Reasons for undergoing malaria tests, the results of the tests and testing facilities were not investigated. Few (4.2%) of the respondents were reportedly infected with malaria within the period under investigation. Detailed characteristics of the respondents are presented in Table 1.

**TABLE 1:** Demographic characteristics and malaria experience of respondents from Bushbuckridge from August 2007 to July 2008.

Characteristics	Respondents					

	All†		Male‡		Female§	
		
	Frequency	%	Frequency	%	Frequency	%
**Age in years**						
< 30	196	32.6	49	32.5	147	32.6
30–39	136	22.6	32	21.2	104	23.1
40–49	97	16.1	22	14.6	75	16.6
50–59	76	12.6	21	13.9	55	12.2
60 or more	52	8.6	22	14.6	30	6.7
Unknown	45	7.5	5	3.3	40	8.9
**Highest level of education attained**						
No education	124	20.6	23	15.2	101	22.4
Primary education	95	15.8	20	13.2	75	16.6
Secondary education	353	58.8	100	66.2	254	56.3
Postmatric qualification	15	2.5	5	3.3	10	2.2
Other	14	2.3	3	2.0	11	2.4
**Relationship to the household head**						
Household head	273	45.3	87	57.6	186	41.2
Spouse	88	14.6	3	2.0	85	18.8
Daughter or son	170	28.2	51	33.8	119	26.4
Grandchild	23	3.8	7	4.6	16	3.5
Parent	26	4.3	0	0.0	26	5.8
Other	22	3.7	3	2.0	19	4.2
**Occupation**						
Unemployed or housewife	403	66.9	78	51.7	325	72.1
Farmworker	6	1.0	0	0.0	6	1.3
Civil servant	29	4.8	15	10.0	14	3.1
Private sector	28	4.7	13	8.6	15	3.4
Entrepreneur	7	1.2	4	2.6	3	0.7
Pensioner	61	10.1	20	13.2	41	9.1
Student	38	6.3	15	9.9	23	5.1
Other	30	5.0	6	4.0	24	5.3
**History of malaria testing during the period of August 2007 – July 2008**						
Tested for malaria	69	11.5	7	4.6	62	13.7
Not tested for malaria	524	87.0	144	95.4	380	84.3
Forgot	9	1.5	0	0.0	9	2.0
**History of malaria infection during the period of August 2007 – July 2008**						
Had malaria infection	25	4.2	3	2.0	22	4.9
No malaria infection	572	95.0	148	98.0	424	94.0
Forgot	5	0.8	0	0.0	5	1.1

*Source*: Authors’ original data

† Total number of all respondents is 602 (100%).

‡ Total number of male respondents is 151 (25%).

§ Total number of female respondents is 451 (75%).

### Knowledge of malaria and information, education and communication

The study found that about 93% (95% CI: 90.4–94.7%) of the respondents had heard about malaria, of whom 15.4% (95% CI: 12.5–18.7%) did not know that malaria is caused by a mosquito bite. The results showed no gender or age specific differences in respondents’ knowledge of the causes of malaria (Table 2). Notably, respondents (7.5%) who did not know their ages were not far less knowledgeable about malaria causes than those who knew their ages. All respondents (100%) with tertiary education and 71.8% with no formal education knew that malaria is caused by mosquito bite (Table 2).

**TABLE 2:** Knowledge of malaria causes stratified by gender, age and the highest level of education attained.

Strata	*n*	Frequency	%
**Gender**			
Male	151	120	79.5
Female	451	364	80.7
**Age groups (years)**			
< 30	196	171	87.2
30–39	136	110	80.9
40–49	97	81	83.5
50–59	76	58	76.3
60 or more	52	32	61.5
Unknown	45	32	71.1
**Level of education**			
No formal education	124	89	71.8
Primary education	95	63	66.3
Secondary education	354	305	86.2
PostMatric	15	15	100
Other	14	12	85.7

*Source*: Authors’ original data *n*, given as a means of number.

**FIGURE 4:**
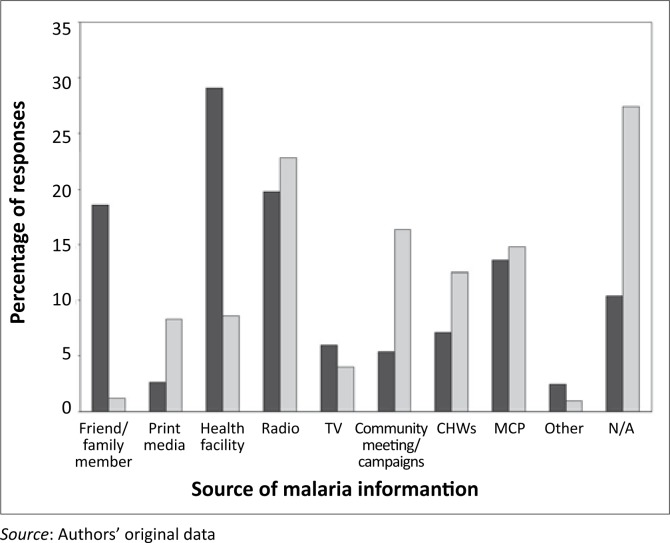
Trends in the difference between the preferred sources (grey bars) of malaria information and the actual sources (black bars), in Bushbuckridge (*n* = 602). CHWs denote Community Health Workers, TV stands for Television, MCP for Malaria Control Programme and N/A for Not Applicable.

The respondents’ sources of malaria information were mainly health facilities (29.1%, 95% CI: 25.5–32.9%) and radio (19.8%, 95% CI: 16.7–23.2%). Malaria communication routes of choice were radio (22.8%, 95% CI: 19.5–26.4%), MCP (14.8%, 95% CI: 12.1–17.9%) and Community Health Workers (CHWs) (12.5%, 95% CI: 10.0–15.4%). More than one-quarter of the respondents (27.4%) did not mention any preferences, as shown by ‘not applicable’ (N/A). The proportion of the respondents who preferred community meetings and awareness campaigns to communicate malaria information, was sizeable (16.4%, 95% CI: 12.4–21.6%), which mirrored characteristics of a close-knit community (Figure 4).

**FIGURE 5:**
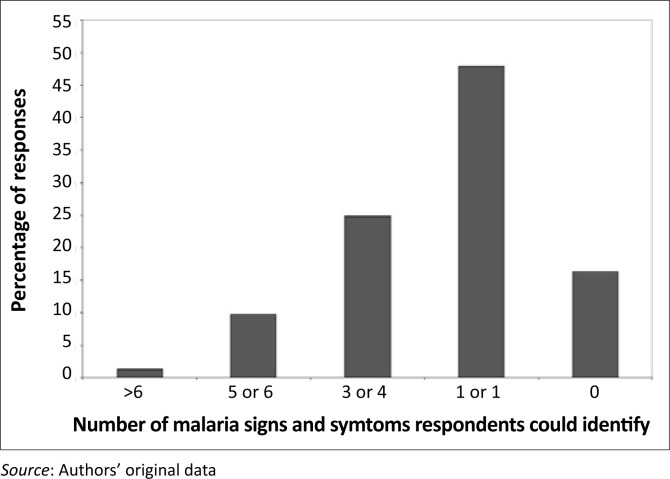
The number of malaria signs and symptoms as identified by the respondents (*n* = 602).

**FIGURE 6:**
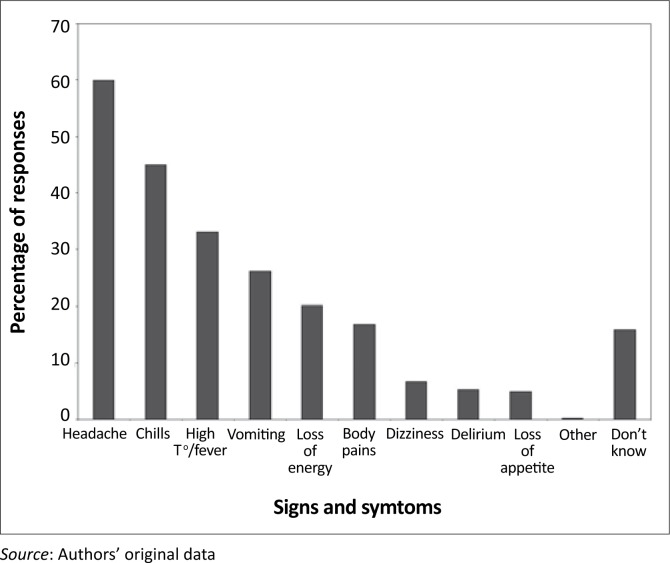
Respondents’ knowledge of malaria signs and symptoms (*n* = 602) with respondents allowed to list as many signs and symptoms as possible.

Notably, most people (88.2%, 95% CI: 85.3–90.6%) were keen to know more information about malaria, and 90.7% (95% CI: 88.0–92.8%) believed that malaria can kill if it is untreated. This keenness and attitude towards the fatalistic nature of malaria were interesting findings, especially when viewed against the background of their limited knowledge of malaria signs and symptoms. About 16.2% could not mention even one sign or symptom of malaria, whilst 48% only knew one or two signs and symptoms. Few respondents could identify more than four signs or symptoms of malaria (Figure 5). The most commonly recognised signs and symptoms of malaria (Figure 6) were headache (60%), chills (45%) and high temperature or fever (33%).

### Treatment-seeking behaviour and prevention

**FIGURE 7:**
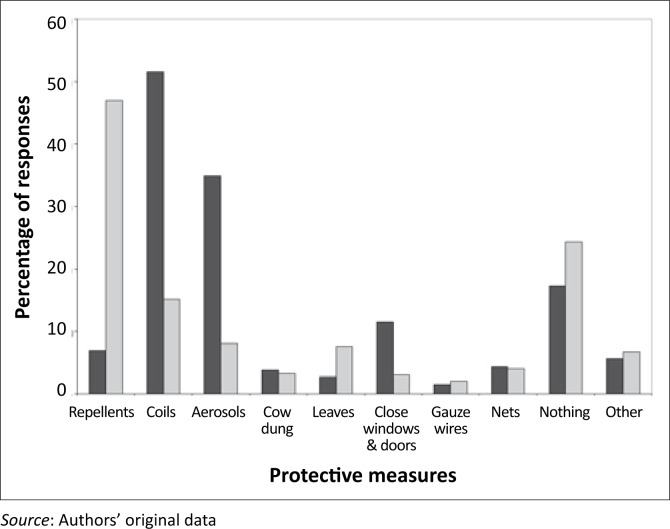
Personal protective measures taken to make house safer from mosquitoes (black bar) as opposed to the measures taken to avoid mosquito bites (grey bar) in Bushbuckridge (*n* = 602).

The study revealed that about 99% (95% CI: 97.5–99.5%) of the respondents preferred to seek treatment at health facilities, 82% of whom intended to do so within 24 hours of the onset of malaria symptoms. Early treatment-seeking intention amongst women (83.6%) was slightly higher compared to that amongst men (75.5%); this trend was unaffected by age. In 97% of the time, those who would delay to seek treatment were unlikely to apply any alternative treatment in the meantime. About 91% (95% CI: 88.6–93.3%) of the respondents believed that malaria can be prevented through various protective measures. Most respondents would normally use mosquito coils (51.5%, 95% CI: 47.4–55.5%) and aerosols (35.0%, 95% CI: 31.3–39.0%) to make their houses safer from mosquitoes, as well as repellents (47.0%, 95% CI: 43.0–51.1%) to avoid mosquito bites (Figure 7). A substantial number of respondents were unlikely to apply any preventive measures to make their houses safer from mosquitoes (17.3%, 95% CI: 14.4–20.6%) and to avoid mosquito bites (24.3%, 95% CI: 20.9–27.9%) (Figure 7). Of the 17.3% (*n* = 104) who would do nothing to make their houses safer from mosquitoes, 75% (*n* = 78) reported that their houses had been sprayed within the previous 12 months. About 66% (*n* = 96) of those who would do nothing to avoid mosquito bites have had their houses sprayed during the previous spray round. The low use of mosquito nets reflected the non-availability of a policy on the free distribution of bed nets at all levels in South Africa, that is, at subdistrict, district, provincial and national levels.

### Indoor residual spraying

The results showed that MCP is well known (99.5%, 95% CI: 98.4–99.9%) by the people of Bushbuckridge, who mainly associated its functions with indoor residual spraying (IRS) activities. About 69.8% (95% CI: 65.9–73.4%) of the respondents reported that their households had been sprayed, 27.6% (95% CI: 24.1–31.4%) had not been sprayed and 2.7% (95% CI: 1.6–4.4%) did not know whether their households had been sprayed during the previous spraying season. Re-plastering amongst the 420 (69.8%) sprayed households and 166 (27.6%) unsprayed households was 6.7% and 4.2% respectively (Odds Ratio = 1.62). The two main reasons for nonspraying of certain households were firstly that no one came to spray, and secondly that no one was at home at the time of spraying. Most respondents (87%, 95% CI: 84.0–89.6%) reported knowledge of the reasons for spraying and correctly identified the ‘killing of mosquitoes’ as the key reason. However, 29.4% of the respondents did not like certain aspects about spraying, especially the discolouring of inner house walls by the insecticide (Table 3).

**TABLE 3:** Aspects that people dislike about spraying in Bushbuckridge.

Reason	Frequency (*n* = 177)	%
Insecticide has an unpleasant smell	46	26.0
Inconvenience	4	2.3
No malaria (few cases)	18	10.2
Invasion of privacy	2	1.1
Excites other insects	13	7.3
Damage to belongings	27	15.3
Spraying person's conduct	28	15.8
Discolouring house walls	49	27.7
Other	11	6.2

*Source*: Authors’ original data *n*, given as a means of number.

## Discussion

### Knowledge of malaria

Unlike many malaria-endemic-prone and malaria-epidemic-prone settings in Africa,^14, 33^ appropriate knowledge of the causes of malaria was relatively good in Bushbuckridge. However, sustaining community knowledge of malaria and its causes may be severely challenged, and even compromised by the degree of cross-border migration between Mozambique and Bushbuckridge.^15, 28, 29^ Studies have noted that improved community knowledge of malaria and its source of transmission promote preventive and personal protective practices amongst the affected populations.^9, 15^ Contrary to the results of a study conducted in Tonga, Mpumalanga,^34^ which established a close association between knowledge of the causes of malaria and disease symptoms (*p* < 0.001), no firm relationship between the knowledge of malaria causes and disease symptoms was identified in this study. The results show that inadequate knowledge of signs and symptoms in Bushbuckridge does not appear to threaten intentions of prompt treatment-seeking practices at this stage. However, if no attention is paid to educating the community on malaria signs and symptoms, the future implications cannot be guaranteed because other studies have established that the knowledge of signs and symptoms plays a particular role on early diagnosis and treatment.^35^

### Malaria information, education and communication

Congruent with other findings,^2, 36^ this study identified radio, as the medium for spreading information speedily and widely, as one of the most commonly used malaria information communication routes. The number of respondents who preferred CHWs and MCP to communicate malaria information was noteworthy if it is taken into account that CHWs have been found to be effective in delivering messages on specific control, treatment and prevention behaviours on individual bases.^39^ The use of community meetings and awareness campaigns in this study is indicative of the strength and value of involving community leadership and structures as strategic partners in malaria-control activities. Without community consent and cooperation, for example, successful implementation of interventions such as IRS will be an illusion. It is, therefore, necessary to ensure that locally acceptable and appropriate communication routes are used for information, education and communication (IEC) interventions. There are serious concerns however, about the fact that 27.4% did not specify any preferred information communication routes, and this finding requires further investigation. Despite mixed responses on the respondents’ preferred malaria information communication routes, their attitude and keenness to receive more information about malaria was positive.

### Treatment-seeking practices in terms of promptness and treatment options

Respondents’ intentions to seek treatment promptly and their choice of a treatment facility in Bushbuckridge exceeded the Roll Back Malaria (RBM) recommendation, which states that at least 80% of those infected with malaria should seek prompt (within 24 hours of the onset of symptoms) treatment.21 Similarly, the Lubombo Spatial Development Initiative (LSDI) region, a collaborative approach to malaria control between Mozambique, Swaziland and South Africa, was found to have high prompt treatment-seeking habits.^38, 39^ This trend was contrary to many African countries^13, 14, 40, 41, 42^ whereby nonpublic health facilities are mostly preferred and promptness remained as low as 13–30%.^14, 40, 42^ Treatment-seeking trends in LSDI were accounted for by the effectiveness of country MCPs and the project successes.^43^ The inhabitants’ willingness to seek treatment promptly in Bushbuckridge was explained furthermore by their attitude towards malaria, whereby over 90% thought that malaria can be deadly if left untreated. It should be noted that a positive attitude is particularly important when attempting to change behaviour.

### Personal protection against malaria and indoor residual spraying

Most respondents in this study correctly distinguished between measures intended to make a house safer and those intended to avoid mosquito bites. Of more concern was the fact that between 17% and 25% of the people did not use any personal protection against malaria. On a somewhat positive note, 66–75% of those who did not use any personal protection were protected by IRS, as they reported to have had their houses sprayed during the previous spraying round. However, the remaining 25–34% whose houses were not sprayed and yet did not use any personal protection was equally concerning. Mosquito net ownership and use was low, probably because South Africa does not have a mosquito nets distribution programme. The results of this study suggest that IRS coverage still need some improvement, especially as WHO recommends a minimum of 80% spraying coverage for IRS to be effective.^32^ Re-plastering as a common social practice identified by many studies to compromise the residual efficacy of the insecticide,^44^ was relatively low in Bushbuckridge, whereas the practice was found to be a serious threat in the study conducted in KwaZulu-Natal, South Africa.^45^

It is very interesting that this study has shown that re-plastering in Bushbuckridge is often undertaken to prepare for the festive season, as opposed to the dominant thinking that people solely re-plaster to remove insecticide marks on the house wall.^45^ Even more interesting was the fact that the proportion of householders who re-plastered after spraying did not enormously differ from those whose households were not sprayed but re-plastered anyway. This finding suggests that there is more to re-plastering than just a reaction to insecticide marks on the walls; hence interventions directed at discouraging re-plastering should take this factor into consideration and should probably advise the community on the acceptable time of the year to re-plaster their houses. This recommendation takes into consideration the fact that it may be an ambitious view to believe that re-plastering could cease completely.

## Conclusion

In conclusion, most respondents had a fair knowledge of malaria and its transmission. Educating communities on malaria signs and symptoms may need some attention before it negatively affects treatment-seeking behaviour. In line with WHO recommendation, IRS coverage needs some improvements, especially because it remains the mainstay of vector control intervention in Bushbuckridge and other malarious areas in South Africa. It is apparent that a multiplicity of factors are involved in the decision to re-plaster which suggests that new strategies and approaches are required to address the impact this practice has on the effectiveness of malaria control efforts. The results of this study displayed generally acceptable community awareness on effectiveness of malaria control efforts. However, the fact that the study did not investigate the respondents’ origins and their migratory history is considered a limitation, especially given the degree of cross-border movement between Mozambique and Bushbuckridge. The impact of migration on malaria in Bushbuckridge was not adequately explored either.
